# Between Gift-Giving and Accumulation: Peer Review Economies in Psychology

**DOI:** 10.1007/s11024-025-09582-2

**Published:** 2025-05-28

**Authors:** Wolfgang Kaltenbrunner

**Affiliations:** 1 Centre for Science & Technology Studies, Leiden University, P.O. Box 905, 2300AX Leiden, The Netherlands; 2 Research on Research Institute, Leiden, The Netherlands

**Keywords:** Peer review, Psychology, Gift economy, Accumulation, Scholarly communication

## Abstract

Peer review is crucial for academic communities to ensure high-quality research, but yields relatively limited formal rewards for the individuals who perform it. Drawing on 39 semi-structured interviews, I study how reviewers for three publishing outlets in psychology experience the tension between community responsibility and various priorities of a more individual kind. Peer review functions as a gift economy, in which authors are expected to repay the work reviewers have invested in their submission by providing peer review for others. This gift economy is embedded in other economies, which are based on the accumulation of various types of capital (recognition, visibility, money etc.). If not kept in check, the competitive dynamics of accumulation tend to undermine the sense of reciprocity required for the continued functioning of peer review. One of the journals studied here is particularly prestigious and operates with a traditional format of pre-publication peer review. The model effectively exploits the tensions between gift giving and accumulation. It exposes reviewers to various undesirable submission practices characteristic of high impact publishing, but compensates them with a share of its reputation that reviewers can use to advance their own careers. The other two publishing outlets are based on not-for-profit business models and feature elements such as open peer review, portable reviews, and registered reports. Reviewers can thereby more easily see their work as something akin to co-authorship. This alleviates tensions between gift giving and the various encompassing economies, contributing to the sustainability of the respective peer review systems.

## Introduction

Recent debates about improving peer review in a scholarly publishing context are often focused on questions of its efficacy in detecting fraud and questionable practices; on concern with avoiding bias and ensuring fairness in the hierarchical relation between authors and reviewers; and also on the challenge of broadening participation in peer review to ensure that a diverse set of opinions is represented (Waltman et al. 2023). In this paper, I instead approach peer review from a fourth important vantage point, namely as a matter of economic organization and incentives.

Peer review is, on the one hand, crucial for academic communities to ensure high-quality research. At the same time, it yields relatively limited formal benefits for the individuals who perform it. Commonsensical knowledge among academics as well as sociological studies suggest that researchers carefully manage their working hours under the aspect of career advancement, and that they subordinate many choices in their everyday practice to the priorities of publishing and grant acquisition (Nastesjö 2024; Kaltenbrunner et al. 2022; Rushforth et al. 2019). This raises the question of how such concerns of a more individual nature can be reconciled with the communal labor required for peer review - the demand for which is rising proportionally to the volume of submissions to journals. Widespread complaints about delays in peer review and the difficulty to recruit suitable reviewers in the first place suggest the issue has recently become more pronounced, arguably because of growing levels of competition for funding and employment more generally. The underlying tension is of course a more basic and arguably foundational problem for the organization of scientific work. Scientists carry significant individual risks and can earn equally significant accolades in case of success. Yet their individual achievements usually depend heavily on less explicitly recognized forms of communal labor, for example, to maintain shared infrastructure, to enable complex forms of scientific collaboration, and to ensure mentoring and supervision (Hagstrom 1965; Traweek 1988; Ziman 2000).

In this paper, I investigate how three different journals and publishing platforms in the field of psychology manage the tension between communal labor for peer review and more individual priorities of participating actors. Psychology is a timely case study because of significant variation in publishing and review models. Such variation has been fueled by a so-called replication crisis that has threatened to undermine the legitimacy of the field in the perception of the wider public (Yong 2015). Almost ten years ago, an influential meta-study in *Science* showed that a significant number of publications in leading psychology journals is not reproducible (Open Science Collaboration 2015). As a reaction to this debate, we have seen significant experimentation with novel publishing workflow features, such as preregistration or various forms of open peer review. These arguably have strong implications for the work of reviewers, for example in the sense of defining certain expectations towards their evaluative practices and sometimes also by reconfiguring the role of this work in the larger publishing workflow.

Below, I will first introduce a theoretical framework in which I propose that peer review can be conceptualized as a gift economy, based on reciprocal exchange of favors among members of a community. However, such gift exchange is constantly threatened by other imperatives in scholarly publishing that are rather based on a logic of accumulation of different forms of capital, including scholarly recognition and commercial profits for publishers. I will then give an overview of the current state of publishing models in psychology, followed by a description of my case studies and data collection. The main part of the paper is an analysis of how the particularities of different publishing models – one a more traditional journal based on pre-publication peer review, the other two publishing outlets with several more unconventional features - configure the inherent tension between gift-giving and accumulation, and what this means for the continued willingness of reviewers to donate time for peer review.

### Gift-giving and Accumulation

In this paper, I build on an earlier argument according to which peer review systems can be conceptualized as gift economies (Kaltenbrunner et al. 2022; see also Hagstrom 1982). Inspired by the work of anthropologist Marcel Mauss (2016), but compatible also with social exchange theories (Cropanzano et al. 2017), this denotes a system of trading favors on the expectation of reciprocal compensation. Authors are thus implicitly expected to repay the work community members including editors and reviewers have invested in their submission by in turn providing peer review for others in the future. In the early phase of a journal, perceptions of reciprocal loyalty will often be more personal, since the community of readers and authors is smaller. In the case of more established publication outlets, mutual obligation will tend to be experienced more towards the journal as an institution or also towards the communities and sub-communities it is now seen to represent. At the same, the sense mutual obligation can never be taken for granted and instead is a perpetual project to strive for (Treviño 2008). Individual perception of fairness and proportionality is key here. For cycles of gift exchange to sustain themselves long-term, researchers must feel that their investment of time and effort is proportionally reciprocated. The gift exchange typically requires some coordination by editors, for example to ensure intellectual fit and mutual respect in the exchange between authors and editors.

Yet the peer review gift economy does not exist in a vacuum. It is embedded in other economies, which function more on the basis of accumulation of various types of capital. In what I call the scholarly reputation economy, publications are central (Fochler 2016; Hessels et al. 2019). Scholars who publish a lot in prestigious journals tend to be associated with their research topics and – where their work is taken up by peers - build up recognition that has various performative effects, for example that of increasing the chance to attract future funding and citations. Accumulated prestige can also be deployed at specific moments in a career, for example when researchers apply for academic positions. Peer review obviously plays an important enabling role in this reputation economy, insofar as an effective peer review system is an important precondition for journals to function.

The landscape of scholarly publishing outlets can itself be seen as an economy. Academic communities have grown and given rise to a large range of journals and publishing platforms. The competition for visibility to academic audiences prompts editors to more intentionally position publishing outlets vis-à-vis each other, for example by crafting a distinctive scientific profile and by ensuring indexation in various databases and citation indexes (Wang et al. 2023). A particularly powerful differentiation is the hierarchy created by the journal impact factor. Journals with high impact factors promise a particular form of reputational gain for researchers who publish in them, which will affect also their ability to recruit reviewers. Again, journals can only continue to claim their position as important outlets vis-à-vis competing journals if they have an effective peer review system in place, that is, a large enough pool of committed reviewers that are ready to deliver reviews on time.

Of relevance to this analysis is, lastly, the economy of publishing as a business. Scholarly publishing is a multi-billion-dollar industry populated by a wide range of actors who pursue equally diverse commercial strategies. Some, like university presses or scholarly society publishers, aim to break even or achieve modest economic gains, while large commercial actors such as Elsevier or Springer constantly aim to increase their already considerable market shares and defend their oligopolistic position (Brainard 2021; Posada & Chen 2018; Pinfield et al. 2020). Again, peer review plays an important role in enabling this economy. To clarify it, let us review a simple equation to calculate the operating margin (OM) of publishers, as proposed by Vann (2017). The operating margin is “the money remaining after subtracting from sales revenue (SR) the costs for operating expenses (OE) and cost of goods sold (COGS)” (OM=SR-[OE+COGS]). OEs here include managerial and admin functions, marketing, and asset depreciation; while COGS are costs incurred by the production of articles, incl. writing, reviewing, editing, copyediting etc. For operating margins to grow, SR need to increase while COGS – including peer review, arguably the second most laborious task after writing an article - need to be stabilized. A recent study estimates the number of hours spent by reviewers globally at 100 million hours in 2019, which translates to an economic value of at least 1.9 billion USD (Aczel et al. 2021). In other words, financially uncompensated review labor plays a crucial role in sustaining the economic profitability of publishing as a business.

In many ways, the embedding of the peer review economy in the various other economies can be quite functional from the perspective of research communities interested in high quality reviews. For one, researchers’ interest in gaining reputation to some extent incentivizes reviewing. Researchers may, for example, seek community recognition by participating in peer review for prestigious journals, and reputed authors are often asked to join editorial boards and thereby take on a particular review workload and communal responsibility (Whitley 2000; Zaharie & Osoian 2016; Ohly & Schneijderberg 2022).

But also the embedding of the peer review gift economy in publishing as a business can be functional. The unpaid work of reviewers can, for example, be facilitated where it is effectively supported by commercial actors with suitable infrastructural tools that would be difficult to afford for smaller-scale, not-for-profit publishers (Table 1).

**Table 1:** Number and role of informants

Publication outlet / Number of informants	PS	Collabra	PCI:RR
Reviewers	12	9	14
Managing editors/recommenders	1	0	1
Editors-in-chief	0	1	1
Total number	13	10	16

Yet the mutual embedding of gift and accumulation logics is also a source of tensions that potentially discourage participation in peer review and degrade the quality of review work. Previous research has amply demonstrated some of the negative side effects of the pronounced competition for recognition and visibility in scholarly publishing (Aczel et al. 2021; LeBlanc et al. 2023). An almost universal experience of reviewers is that their contributions are not considered an important achievement in academic hiring and appointment processes, which incentivizes them to prioritize their own publishing over performing peer review. Yet there are also less immediately obvious conflicts. The process of doing research is by definition underdetermined and exposes researchers to significant risk of failure, for example when a project ends up producing results that are not considered interesting by peers, or when it takes very long to produce publishable results. This can become career threatening especially for researchers in precarious positions (Sigl 2012; Hackett 1990). In order to reduce the risks, authors may feel encouraged to approach the review process more as a game of chance. They may, for example, venture a submission to test the reaction of reviewers, even where they are aware of weaknesses in their work. Some authors may also be intentionally dishonest in how they present their arguments. Such submission practices tend to go at the cost of review labor. Underdeveloped and otherwise problematic manuscripts often require more attention by reviewers (cf. Stewart 2025), and when they are rejected, it is common practice to immediately resubmit them to other journals, thus initiating a separate review process that again takes up a significant amount of time and effort (Casnici et al. 2017).

The accumulation-based reputation economy thus produces not just successful research, but also a disproportionate overhead workload especially for editors and reviewers, which is often perceived as wasteful (Tennant 2018; Bell & Kjavo 2018). Such efficiencies can be exacerbated by particular editorial strategies. According to a common complaint, journals sometimes fall victim to so-called publication bias when trying to improve their position in the publishing landscape (Nosek et al. 2012; Kühberger et al. 2014; Nelson 2020). In this view, editors and reviewers are thought to give preference to contributions that they think will draw attention to the journal, which in turn creates additional incentives for researchers to overstate the significance of their findings or even engage in outright misconduct.

There is also a tension between gift giving and publishing as a business that can indirectly degrade the conditions for high-quality review work. Donating time for peer review implicates researchers in commercial business strategies that have long been problematized by critical observers. A well-documented practice is that the four largest commercial publishers pursue a strategy of so-called big deals, that is, bundling subscriptions to a large number of journals in a single package. University libraries thereby lose the ability to pick and choose journals that are most relevant to their audiences, and to negotiate more favorable prices for individual journals (Bergstrom et al. 2014; Pinfield et al. 2020; Beverungen et al. 2012). The ability of publishers to pursue such strategies in turn depends on their market share and the number of important journals in their portfolios (Brainard 2021; Posada & Chen 2018; Pinfield et al. 2020). Where it is donated to commercially operated journals, unpaid academic work for peer review thus can be seen to contribute to undermining the budgetary resilience of academic institutions. Strained university budgets in turn contribute to deteriorating working conditions for many researchers and threaten also the preconditions for producing careful and constructive reviews (time, attention, curiosity etc.). To be sure, for a long time, the economics of publishing were not a matter of explicit reflection in academic communities and instead delegated to university libraries who negotiated contracts with publishers. However, recent debates about OA, the politics of participation in science, and publisher oligopolies have created increasing critical awareness among researchers (Pinfield 2024).

The above examples highlight conflicts of economic principles. Gift logic depends on ensuring a constant sense of reciprocity between authors and reviewers. Yet the accumulation-based economies in which it is embedded incentivize various kinds of undesirable individualistic behavior and (re)produce oligopolistic market asymmetries, which in turn exacerbate budgetary challenges for research institutions. These conflicts can result in gift obligations becoming overstretched. For example, where too many researchers prioritize their own publishing over reviewing for a particular journal, the remaining reviewers will have less time and attention to engage in constructive review work. And if reviewers are recurrently confronted with questionable submissions, they may feel that their investment of time and effort is not respected and decide to stop reviewing or gravitate towards a more superficial review style. Gift relationships in peer review can also be disrupted by the overt intrusion of commercial interests. Where article processing charges (APCs) of a journal are too high or publisher business models particularly intransparent, the willingness of academics to perform peer review as legitimately unpaid communal work may break down (Table 2).

**Table 2:** Career stage of informants

Publication outlet / Career stage of informants	PS	Collabra	PCI:RR
Postdocs	1	1	5
Assistant professor or lecturer	3	2	5
Associate or full professor	9	7	6
Total number	13	10	16

Importantly, while the conflicts I theorize in this section are of a structural nature and represent collective action problems that involve entire academic communities, their employing institutions, and publishers (Neylon et al. 2019), the resulting tensions can be managed more or less effectively on the local level of a given publishing outlet. For example, journals can choose different strategies for incentivizing reviewers and for managing the supply of review labor at their disposal. They also have opportunities to moderate submission practices in different ways, namely by promoting particular normative values around publishing and posing formal requirements for submitting authors. Opting to enter or continue commercial relations with publishers and creating transparency about business models similarly is a choice.

In the remainder of the paper, I will offer a comparative analysis of how different journals and publishing platforms deal with the structural conflicts between peer review gift logic and accumulation-focused logics in which it is embedded. I will also interrogate how such different approaches shape the work and outlook of reviewers, and what are the implications are for the sustainability of the respective review systems in the long run.

## Background: Epistemic Culture and Publication Practices in Psychology

In the beginning of the 20th century, the field of psychology was characterized by a plurality of qualitative and quantitative research methods, involving for example detailed qualitative case studies, disciplined introspection, and physiological experiments (Danziger 1990; Flis & van Eck 2018; Flis 2019). At the same time, many psychologists engaged in interpretive theory building on a grand conceptual scale, for example, in the sense of proposing comprehensive explanatory frameworks to understand the origins and functioning of human consciousness (usually strongly informed by contemporary social norms). Yet after the Second World War, the rapidly growing discipline gravitated to an empirically deductive epistemology that placed a dominant emphasis on standardized quantitative methodology built around inferential statistics. Fundamental theoretical discussions about the nature of the mind gradually disappeared and criticism of the assumptions behind dominant research methods – for example, those that underpin the null-hypothesis – became the exception (Greenwald 1975). In the 1960s and 70s, the growth and differentiation of the field continued, with the emergence of new subfields and interdisciplinary formations such as cognitive science. The trajectory of the field can thus overall be described as an ever more firm embrace of scientific values such as methodological exactitude and reduced scope for interpretive reasoning, motivated by the desire to claim some of the authority accorded to biomedicine.

Sociologists of science have demonstrated how the specificities of particular fields such as their relative degree of intellectual and social integration shape disciplinary publication cultures, including styles of decision-making in evaluative settings (Knorr-Cetina 1999; Whitley 2000; Lamont 2009; Hylmö et al. 2024; Laudel 2024; Reymert 2020). This is true also for psychology. As part of the move towards an essentially methods-driven field, psychology researchers largely abandoned writing monographs and today publish predominantly co-authored journal articles that reflect the collaborative nature of their work. The journal landscape is dominated by venues that rely on a traditional model of pre-publication peer review. There is widespread agreement that the publishability of article manuscripts hinges at least partly on showing the comparability and relevance of findings by means of significance testing and testing of effect sizes. This form of judging quality of contributions abstracts from the particularities of individual findings and thereby accommodates two characteristic features of psychology research. On the one hand, it epistemically accommodates the elusive nature of research objects in psychology. The processes studied by psychologists—attention, memory, emotional responses—can, after all, usually not be observed directly. Moreover, it is compatible with the co-existence of many competing research programs in the field, held together by shared research methods rather than widely agreed-upon problem definitions and research priorities.

When it comes to the use of metrics in evaluation and publication choices, psychology can arguably be situated somewhere between biomedicine and economics, one of the more exact social sciences (cf. Hammarfelt & Rushforth 2017). The journal landscape is highly stratified in terms of perceived reputation of publishing outlets, as measured by the journal impact factor. Journal rankings do not play as important a role as in economics, perhaps because there is significantly greater diversity in terms of research priorities across its many subfields (Fourcade et al. 2015; Hylmö et al. 2024). This situation of reviewers and editors presiding over the allocation of scarce prestigious publishing space configures their relationship with authors in a particular way. Not dissimilar to prevalent conventions in economics (Fourcade et al. 2015; Hylmö et al. 2024), reviewers are often seen as gatekeepers, and since reviews are typically anonymous, there are longstanding complaints about reviewers abusing their authority (Schiavone et al. 2022). Authors in turn are often seen in a preemptively suspicious light, namely as trying to get their submissions into the journal even at the cost of questionable data practices designed to inflate statistical significance. Colloquially summarized under the label of p-hacking, these include selective reporting of dependent variables, selectively stopping data collection when statistically significant results have been achieved, excluding outliers, other practices (Stefan & Schönbrodt 2023; Yarkoni 2022).

The debate about the replication crisis erupting in psychology in the early 2010s is intimately connected to a critique of the incentives created by a reputationally highly stratified journal landscape (Yong 2015). Critical observers in the field began to associate questionable research practices with a perceived tendency of journals to prefer manuscripts with positive and ideally strikingly counter-intuitive results that draw attention. The replication crisis has also heavily informed how psychology researchers have practically interpreted the discourse around Open Science (Tennant et al. 2020). While for other fields, questions around accessibility and the politics of participation were a driving force, Open Science innovations in psychology are widely seen under the aspect of facilitating reproducibility of publications and transparency in the publication process.

A first recent innovation has been an explicit focus of some journals on the soundness and rigor of submissions, meant to limit room for subjectivity in judging the topical interest or novelty of a submission (Spezi et al. 2018; see also BMC Psychology, n.d.). Often combined with a requirement to openly share the data underlying a submission, this signals to authors that reviewers will specifically watch out for questionable data practices and inflated claims.

Another response has been the rise of preregistration (Jonas & Cesario 2015; Lakens 2019). Mainly used for hypothesis-testing research, this publishing formats entails that authors register their expected research outcomes before analyzing data. The aim is to thereby prevent researchers from retrospectively adapting their hypotheses to unexpected findings in an effort to inflate their real significance. Ideally, it also reduces the extra work reviewers otherwise have to invest to detect such practices. A special form is registered reports, where authors have the opportunity to submit a research design and receive reviews reports before carrying out any research (Nosek & Lakens 2014; Chambers & Tzavella 2022).

Another response has been the partial adoption of open peer review, which means that review reports are publicly available and sometimes also signed. Open peer review is associated with many different benefits and downsides (Ross-Hellauer 2017; Wolfram et al. 2020; Collabra: Psychology, n.d.), but in particular with Wolfram et al. additional accountability in peer review and ensuring careful reviews.

Preprint publishing is another pertinent innovation. In contrast to the traditional model of review-then-publish, it allows researchers to circulate papers in parallel to or also instead of submitting them to a journal. While this at first sight runs counter to the goal of enhancing scrutiny of manuscripts, preprints remove barriers to access and can subsequently be reviewed on dedicated platforms. Not least, preprints decouple the publication process from any real or perceived publication criteria wielded by journals (Stojanovski & Marušić 2024; PCI:RR, n.d.).

Portable or transferable reviews are an innovation that aim to decrease pressure on peer review systems arising from competition for limited publishing space (Waltman et al. 2021; PCI:RR, n.d.). A common practice of authors nowadays is to immediately resubmit rejected manuscripts to other journals, thereby restarting a separate review process. Authors will often repeat the process until the manuscript gets accepted. Portable reviews instead allow for review reports to be forwarded to journals alongside a submission, thereby reducing the amount of additional review work that is required. Portable reviews also mean that mutual obligation dynamics are no longer mediated by particular journals as an organizing principle.

Finally, the growing diversity of publishing business models in psychology and other fields is worth mentioning (Mellins-Cohen 2024). Legacy commercial publishers typically operate with a combination of both subscription fees for academic libraries and APCs. Yet many journals and publishing platforms have begun experimenting with alternative business models, for example in the sense of introducing comparatively low APCs, creating transparency regarding the use of APCs by the publisher, or various kinds of non-profit business models.

### Case Studies & Methods

In this paper, I focus on three publishing outlets that combine elements of the above publishing workflow features in different configurations. *Psychological Science* (PS) is a long-established journal operating with pre-publication peer review. PS is the official journal of The Association for Psychological Science and commercially operated by SAGE. By virtue of its high impact factor and historical visibility, PS offers a particular widely recognized form of prestige for authors across all subfields of psychology. Very recently, a number of innovations were introduced in its review workflow, including an encouragement and later a requirement to make raw data accessible, as well as the opportunity to preregister (Association for Psychological Science 2023; Bauer 2022). However, these recent innovations are not yet apparent in the empirical material collected for this paper.

*Collabra: Psychology* is a more recently founded journal with an explicitly broad outlook, both in terms of thematic focus of contributions and regarding the type of publications (original research articles, articles presenting more incremental contributions, replications etc.). The journal has been assigned a journal impact factor in 2022. Its publishing workflow entails that review reports are published alongside articles and can optionally be signed. Its acceptance criteria explicitly prioritize robustness of methods over conceptual novelty of submission (Collabra: Psychology, n.d.), and authors are required to make their data openly available. Collabra is operated by the University of California Press on the basis of comparatively low APCs. The journal website documents the exact use of the charges in significant detail.

*Peer Community In: Registered Reports* (PCI:RR) is a platform for reviewing registered reports. This first involves publishing a research design as a preprint and submitting it to PCI:RR, which organizes a peer review process. Once the design is approved by reviewers, authors enjoy in-principle-acceptance, meaning that the final submission will be published regardless of the outcomes, as long as it adheres to the original design. Authors can choose to submit their reviewed papers to a number of collaborating journals. So-called “friendly” journals publish preprints recommended by PCI:RR as they are, whereas “interested” journals consider PCI:RR reviews but potentially commission additional ones. While PCI:RR is principally open to the submission of registered reports from across all fields, current publications and the list of collaborating journals show a strong focus on psychology. The publishing model of PCI:RR is exclusively based on a combination of subsidies and is completely devoid of commercial interests (PCI:RR, n.d.).

Between March 2023 and April 2024, I have conducted 39 interviews with reviewers and researchers in various editorial functions, with interviews ranging between 25 and 50 minutes. For both Collabra and PCI:RR, I have relied on optionally signed review reports to contact potential informants. For PS, I have used the 2021 thank-you-letter to reviewers, in which reviewers are mentioned by name (Bauer 2021). In all cases, I have aimed for gender balance and balance in terms of academic seniority (at the time of reviewing).

My interview questions focused on the scientific background of the informant, their opinions about different publishing and review models, their outlook on peer review in general, and more specifically on reviewing for the publishing outlet in question. Having transcribed the interviews with the help of software, I analyzed them through iterative coding in Atlas.ti. The broad outlines of the analytical categories were derived from a preexisting theoretical interest developed in a previous research project (Kaltenbrunner et al. 2022), namely the idea that peer review functions as a gift economy but is potentially undermined by disruptive dynamics in the research environment. This initial set of categories was iteratively refined to better capture the empirical specificities of the case studies at hand, in particular differences between publishing and review workflows.

### A Balance of (Dis)incentives - *Psychological Science*

When informants talked about their likelihood to accept review assignments, most tended to refer to traditional pre-publication peer review as a default, as outlined at the beginning of the previous section. Many respondents explained to be operating with some sort of ethos according to which one is obliged to review if one is also publishing, and some also offered rather precise estimations regarding the desirable ratio of reviews-to-articles. A number of informants suggested one should do at least twice as many reviews for every article published “to keep [your] footprint null” (PCI_14), while another suggested that a ratio of one-to-one is acceptable (PDm_PS_4). Informants explained that they had consciously adopted such proportionality reasoning from their supervisors, or found to have been operating with an implicit rule of thumb when prompted by my question. Estimations such as these give us a very rough idea of the amounts of work researchers overall expend on peer review. Yet they also tend to mask the more intricate workings of the underlying review economy of particular journals, whose sustainability is not just a matter of quantitative proportionality.

A particularly important factor shaping the review systems of journals is their perceived importance in a community. Journals with high impact factors such as PS tend to attract many more submissions than they can easily handle. This includes submissions by authors who don’t know much about the journal. Sometimes, authors also submit to prestigious journals in the hope of making it to the second round, yet without properly crafted manuscripts. Even more significantly, authors regularly also act in intentionally misleading ways. The reviewer quoted in the following felt that especially submissions to high-profile journals in social psychology regularly present strikingly counterintuitive arguments that challenge a preexisting scientific consensus on a given question. Yet often, authors try to create the appearance of such groundbreaking findings more through a combination of stylized narration, underpowered empirical work and selective data use, rather than actually robust evidence.

[Social psychology] was most affected [by the replication crisis]. (…) I also think that it's a field where it's more fancy for… for the outreach. So it's easier to get trapped in the fact that I want to somehow become famous because of these results… some ego problems that still affect science, in my perspective. And (…) to emerge [as a famous scientist] in social psychology, you have to provide frequently counterintuitive results, which is something that science shouldn't reinforce that much in my perspective. (PDm_PS_4)^1^

Reflections on the publication culture of mainstream psychology journals often go along with expressions of frustration in relation to review work. There is a sense that authors’ attempts to place their manuscripts in prestigious journals at the cost of underdeveloped, overclaiming and intentionally misleading work is a sign of disrespect towards their colleagues who donate time for peer review. Some informants stated that as a result of their discontent with the situation, they no longer review for journals who operate with traditional publishing models (APm_COL_4, Ex_COL_3).

In principle, journals have means of curbing problematic submissions practices and thus ease the tension between self-interest of authors and commitment of reviewers to communal work. Particularly important is screening of submissions by editors before they are sent out for review, which can serve to protect reviewers from having to handle clearly unsuitable submissions. To be sure, screening also carries risks, insofar as it can make it especially hard for uncommon types of submissions to get through to the actual review stage. One managing editor still emphasized the importance of screening submissions to ensure that reviewers feel that their investment of time and effort is respected.

I send out about… 30 to 35[% of submissions for review], and about 65% [of submissions are immediate] desk rejects (…) I'm sure you've had this experience right? Like you're a reviewer and you get some paper. And you're like, why is anyone spending time on this? And you can't just say that right? You still feel like you have to go through writing the review. And so I think that's a lot of it is like, let's not use people's time if we know that even if they put the time into it, this still isn't just isn't the kind of research that's going to be publishable here. (APf_PSe_8)

Yet some of the reviewers for PS I interviewed in fact criticized the editorial leadership of the journal for not only failing to consistently screen out overclaiming and otherwise unsuitable submissions, but instead condoning and even encouraging them in the hope of drawing attention to the journal in the past (PDm_PS_4, Pm_PS_9, AssPm_PS_11). In the words of one reviewer:

So when I started out [about eight years prior], *Psych Science* was, was very popular, and was particularly known for publishing quick, having quick turnaround shorter papers (…) And they were usually one study or two experiments. And so you know, they published a lot of flashy stuff, right? The stuff that is now suspect, I would say, sort of unlikely, improbable effects and sort of fancy things that pop science loves, but you know, academics themselves know are probably bullshit. (…) And then when the whole replication crisis hit (…), it kind of lost a chunk of his prestige, it was still high impact, and people are still submitting there. But I think they were seen as the poster child of sort of that type of psychology, you know, fast, dirty, like, cheap sort of science, you know, that is like, speaks to your imagination, but actually isn't, it's like not really science in that sense. (AssPm_PS_11)

Even where they don’t lead researchers to stop reviewing for a journal, questionable submission practices can have other negative effects on the work of reviewers. Most importantly, they create a pervasive sense of suspicion about the intentions of authors. One respondent went as far as to describe typical journal manuscripts in psychology as misleading “advertisements” for the underlying data that must be read with utmost skepticism (PDm_PCIr_16). The generalized suspicion towards submissions can make review practice degrade to a form of gatekeeping, in the sense of becoming an active search for faults in a submission that warrant rejection, rather than approaching it under the aspect of trying to improve the argument. Conversely, where reviewers find that submissions make substantial claims and can actually back them up, it tends to come as a surprise, as one of my informants explained:

So, like, I think this may be the last *Psychological Science* article I reviewed. (…) It completely won me over because I started the article and I thought the abstract wasn't clear and I didn't agree with the literature review. I thought it over claimed and missed some things. And then when I went into the methods, I thought, actually, they're doing something really interesting here. And they've actually...You know, I can see now why they reported the literature they did. I wouldn't have pitched it like that. (…) But the work is really good. And so I got to the end of the paper. Quite unusual. But normally you form a judgment and it gets reinforced. I got to the end of the paper and I was like, oh, this is much better than it's written up as. (Pm_PS_13)

What, then, keeps researchers reviewing if negative side-effects of high-impact-publishing constantly undermine the sense of respect and mutual obligation between authors and reviewers? An important part of the answer appears to be the same reason as the one that encourages poor submission practices in the first place, namely, visibility and reputation of the journal. As noted above, PS is a long-established journal with a high impact factor. The brand quality of the journal affects researchers’ likelihood to accept review assignments. Not only is reviewing for a journal like PS a way of staying up-to-date on the literature. It is also a particularly effective way of claiming community membership and an occasion for cultivating relationships with handling editors, which can potentially facilitate prospective submissions of one’s own (cf. Zaharia & Asian 2016). The following quote captures a typical kind of reasoning:

If you're young, you just want to show like, look, I can get my stuff in those journals. It's important because it's so difficult to evaluate your quality. So you'll take any proxy for it that you can. And that’s the same with reviewing, like I would write down like who I have reviewed for and then if there was *Psychological Science* there, I'm pretty sure the first time or *Nature* or something, or *Science*, the first time I got a review request from those journals, I probably accepted them. Because I thought, wow, wow, they're asking me, poor old little me, for this. So I think they drive, they have this kind of [reputation-based] mechanism in addition to the give-and-take and I-submit-and-you-submit. (APm_COL_4)

The above quote emphasizes the importance of reviewing for established journals for junior researchers. Yet there are other groups of researchers for whom the ability to partake in the reputation associated with an established journal like PS by means of reviewing is particularly important. On the one hand, more senior researchers whose careers are based on traditional journal-centric forms of scholarly communication. A number of such respondents stated that they review exclusively for well-known journals, and that the importance and value of other forms of publishing, such as preprints, is somewhat less clear to them (Pm_PS_2, Pm_PS_6). Conversely, one informant felt that more senior colleagues tend to perceive explicitly Open Science-oriented publishing policies as an attack on legacy ways of doing research in psychology (Pf_Col_9). Not least, researchers in emerging specialties in psychology (Pf_COL_5) similarly appeared to be particularly aware of the importance of publishing in conventionally prestigious journals. Being asked to review for them is a way of conferring legitimacy to their research topic.

In summary, the traditional high-impact publishing format of PS – at least before the various recent innovations to its workflow – does little to ease the contradictions between gift giving in peer review and the competitive principle of reputation building. It on one hand requires a particularly large number of reviewers to handle its significant volume of submissions, and it exposes reviewers to various undesirable submission practices that accompany the competition for prestigious publishing space and that promote a generalized suspicion towards authors. On the other hand, it compensates reviewers with a share of its enduring prestige and visibility, which they can use to advance their own position in the competition for reputation.

### Alleviating Tensions Through Transparency - *Collabra: Psychology*

A common approach for researchers is to predominantly review for journals they have published in themselves or aim to publish in. Mutual obligation dynamics in this sense tend to favor the “installed base” of established journals such as PS, since these are widely known and provide a visibility advantage over less well-known outlets across the community at large. Yet most researchers simultaneously take on a smaller percentage of assignments for journals and platforms they are less familiar with, with concrete estimations of the ratio ranging somewhere around 3:1 or 4:1 (Pm_PS_9, AssPm_COL_8, Lf_PCI_9). There is thus overall a significant amount of work being invested in new publishing formats that gives them a chance to develop functional peer review gift economies and build up more visibility. Some respondents even stated that they have decided to opt out of reviewing for traditional high-impact journals, and now instead review predominantly or exclusively for journals and platforms they perceive as progressive for experimenting with new publishing and review workflows (APm_COL_4, Ex_COL_3).

Among more recently founded journals, Collabra has managed to create a degree of visibility throughout the community. While its impact factor is comparatively low, several editorial board members are renowned for their pioneering role in Open Science initiatives in psychology. One handling editor speculated that the journal audience has therefore been limited to researchers already committed to Open Science policies (Lf_COLe_1). Compared to highly visible journals such as PS, this mitigates the challenge of filtering out intentionally misleading or unsuitable submissions in the screening phase.

The extent to which reviewers are confronted with questionable author practices can moreover be shaped by a journal’s formal submission policies, which in the case of Collabra includes the mandatory inclusion of raw data. Most reviewers I interviewed have critical opinions about conventional approaches to quantifying statistical significance and power analysis, which they feel makes research vulnerable to manipulation (Pm_COL_7, Ex_COL_3). Mandatory data inclusion in turn is widely seen as a signal deterring authors from questionable presentation of data, and as such a reason for many reviewers to take on assignments from Collabra over other journals without such a policy (Pm_COL_2, Ex_COL_3). To be sure, the prospect of checking raw data implies a higher workload for reviewers. Exactly how much higher depends on how well presented such materials are.

It's one thing to have, say, an OSF page where you pre-register and you share all your materials and it's quite another thing to have those materials be stored there in a way that other people can understand. (...) There are certainly people who do it really well and have, say, an aggregate… aggregated data sheet that has sort of like a glossary. Here's what the columns are. Here's what the, you know, all this stuff is and that can be fantastic. Some people really, really do a great job. Some people, it seems to me, dump stuff on OSF and they say, well, it's open. (Lm_PCI_6)

As a result, some Collabra reviewers I interviewed explained that they at best take a quick glance at raw data (Lm_PCI_6, Pf_COL_5). Others, however, appear to place significant emphasis on it (APm_COL_4). One respondent even explained their practice of enforcing such a review in journals that do not have a similar policy. In case of submissions that fail to make their data accessible, they only do only a light-touch review (Pm_COL_2).

Another formal feature that helps mitigate the undesirable effects of competition for publishing space – and thus prevents strain on gift relations - is the journal’s open peer review policy. Open peer review plays an important role in establishing a respectful tone in the interaction between reviewers and authors. Several informants explained how the default practice of publishing (optionally signed) reviews makes their attitude towards peer review subject to public scrutiny. It specifically encourages them to reflect to what extent their own peer review approach has already degraded to gatekeeping and instead encourages them to approach it with greater commitment to constructive criticism and mutual obligation. One respondent gave a concrete example. In a closed review, they explained, reviewers can more easily propose their own publications as relevant literature that the authors should engage with and cite, even where it is not strictly warranted. Such requests can create the impression that reviewers abus their authority. A signed review instead prompts reviewers to provide feedback that is perceived as helpful and substantive not only by authors, but also by future readers. There is likely an performative element to this: The more reviewers make sure to act as ‘critical friends’^2^ rather than gatekeepers, the more reason authors have to be weary of abusing their patience through inappropriate submission practices.

Sometimes (…) as a reviewer you have to recommend your own work and if you also sign that review then you just look like you're an egotistical um sort of person whereas if it's… if the review is not signed um and you accompany the recommendation of your own work with other people's work then you can sort of, um sort of hide in the crowd I suppose um and like I feel um I feel like there's a there's a there's a there's a fine line between um being transparent and open and also just like self-promotion and I don't want to fall on the side of the self-promotion if you know what I mean. (Lm_PCI_6)

To be sure, compared to gatekeeping approach to peer review, open peer review does require somewhat more work to ensure it is constructive. Yet the greater workload required is in part offset by other benefits. In the quote below, a reviewer recounts an instance of a longstanding collaboration with a colleague at another university that emerged from a conversation in open peer review.

I'm not going to take on the review unless I'm going to dedicate the time necessary to it, because I'm going to attach my name to it. And on the other side, as a reviewer, or as an author, rather, I've always really appreciated when people sign their reviews because it's, it's actually sometimes led to collaborations and relationships that I wouldn't have had otherwise. (Pm_COL_6)

Lastly, interviews with Collabra reviewers also illustrate how journal business models can preserve or disrupt the sense of reciprocity required to sustain cycles of gift giving. One Collabra reviewer explained that they initially saw peer review as a form of unconditional community service one is simply obliged to perform irrespective of the outlet. Yet as they became more aware of the underlying economics of publishing, the respondent became increasingly unable to see reviewing for commercially operated journals with high APCs in terms of a gift relationship.

At the beginning, it always felt more like a… Oh, I'm getting invited, so I, you know, I should do this. And because that's also the narrative that a lot of researchers have about reviewing that it's community service. And I feel like learning more about the sort of the political economy of journals and in terms of, you know, what what they do in an academic career and how much they how much the publishers earn. It became clear, OK, there is… there is a certain benefit and researchers for me as a researcher to feel like this is community service because that will incentivize me to do it. (Ex_COL_3)

Collabra, on the other hand, operates with comparatively low APCs at 975USD, no subscription cost, and a policy of transparency for how the APCs are used. More specifically, the journal website explains how 700 of 975USD go directly into funding infrastructure. The rest of the money is put into an APC waiver fund, and none of it is redistributed across the publisher’s portfolio. This is in contrast to a majority of commercial publishers who raise significantly higher charges, yet without any explicit justification or the possibility to track their exact use and associated profit margins. The reviewer quoted above explained that they experience Collabra’s publishing model as a context where the tension between commercial operation and gift logic can still be reconciled, making it easier to justify the continued donation of their time and effort. In a variation of this reasoning, several other informants explained that Collabra’s business model encourages them to accept review assignments since they feel that it is a form of creating value that does not serve any commercial parties (Pm_COL_2, Ex_COL_3, Lf_COLe_1, APm_COL_4).

In summary, when compared to PS, various features of Collabra’s publishing model allow authors and reviewers to engage in cycles of gift exchange that are relatively unburdened by the strain of problematic submission practices. The journal thereby effectively eases the tension between the reciprocal logic of peer review and the more individualistic behaviors incentivized by the competition for scholarly reputation. In turn, the journal’s business model and the way it is communicated make it easier for researchers to think of peer review primarily as a form of community service, rather than as unpaid work that benefits commercial stakeholders.

### Reviewing as a Form of Co-authorship–Peer Community In: Registered Reports

PCI:RR is a similarly recent publishing venue as Collabra. Yet in contrast to the latter, it is not a journal in a traditional sense and does not have a journal impact factor. While this, on the one hand, reduces the volume of questionable submissions, PCI:RR recommenders - the platform’s equivalent of managing editors - reasoned that these factors make the task of ensuring a steady supply of reviewers especially challenging (Pm_PCIr_4, PDm_PCIr_16) . One initially worried about the risk of review invitations by PCI:RR ending up in email spam folders (Pm_PCIr_4). This view is compounded by numerous reviewers who recounted bad experiences with handling submissions for unfamiliar outlets that they retrospectively consider “borderline predatory” (Pf_COL_5). Yet at the same time, PCI:RR has some distinctive publishing features that have earned it a degree of visibility among researchers interested in open science. They include a non-commercial business model based entirely on subsidies, as well as open and portable reviews, based on registered reports. As noted above, PCI:RR also differs from most journals insofar as reviewers and recommenders can actively sign up on the website and indicate their availability for handling a particular submission.

Several of these features directly affect how researchers experience the principal tension between gift giving in peer review and the various accumulation logics at play in publishing. A first one is the platform’s preprint-based non-commercial business model, which preemptively removes the risk that commercial interests disrupt the sense of mutual obligation among reviewers and authors, much like in the case of Collabra. The second is the possibility of reusing reviews created for PCI:RR for subsequent submissions to journals, which is meant to reduce waste of review labor that otherwise comes with serial resubmissions and thus potentially frees up time that factilitates constructive reviews. Some of my informants mentioned that the prospect of reducing reviewer workload through portable reviews and the platform’s business model as complementary motivations (AssPm_PCI_11, PDm_PCIr_16). They saw reviewing for PCI:RR not merely as one outlet for community service among others, but instead as a choice against competing forms of academic publishing. For example, one informant regretted that portable reviews still create a possibility for publishing companies to profit from their donated time (Ex_COL_3). Another explained that they signed up as a reviewer for PCI:RR to offset review work they performed for publishers like Elsevier.

I've heard about [PCI:RR before] and I think I even included my profile over that because I think that... Yeah, it's nice to balance this reviewing for Elsevier, for other organizations that may not share the the approach to scientific publishing (...) So to share my time between different publication models. (AssPm_PCI_11)

The focus on openly reviewed registered reports has similarly strong implications for configuring the perceived tension between gift and accumulation logics. Registered reports entail reviewing a research design as well as a subsequent article. Yet while this two-step process implies a greater workload and therefore a disincentive to take on such assignments, the PCI:RR reviewers I interviewed complicated such reasoning somewhat. Some indeed indicated that registered reports are more work and that they are somewhat more cautious to take it on (Lm_PCI_5, Pm_PCI_7). They also emphasized that the individual parts of a submission are more extensive than in a traditional manuscript submission.

I think the hardest, the most amount of work was just the sheer volume of materials to review because (…) of the amount of methodological detail, the art… these manuscripts are much longer and then you get all the appendices with all the materials and the data and code. (Lm_PCI_5)

To be sure, the second review step can sometimes be a bit lighter, as reviewers should at this point be familiar with the basic premise and methodology of a submission. Still, it adds an additional strain to the process insofar as it entails not just an assessment of the manuscript itself, but also of the correspondence between research design and subsequent research. This requires handling different sets of materials and can be perceived as a somewhat awkward moment where authors and reviewers are reminded of the hierarchy in their relation (AssPm_PS_11). The flipside is that the prolonged and repeated interactions help establish a mutual sense of trust. The reviewer quoted below felt that opting for registered reports shows commitment on the part of authors, since the publishing process is more drawn out and removes some of the flexibility authors would enjoy in a more traditional publication format. As such, authors submitting registered reports deserve some preemptive respect. This perception is a striking contrast to the generalized mutual suspicion between authors and reviewers that sometimes accompanies pre-publication peer review for prestigious journals.

[Y]eah, it is a big task to get through all that [a submission to PCI:RR]. It takes a lot longer than a traditional review. I would do it again because I really, you know, admire the people who actually submit these, like the amount of work that they put into it. And I think it's a good learning experience to see that kind of work (Lm_PCI_5).

Others also stated that reviewing for PCI:RR feels like a particularly meaningful time investment, given that the exchange with authors happens much earlier in the research process and thus at a point when suggestions for redesign and revision are much easier to implement. This is in contrast to legacy publishing workflows, where authors are tempted to re-submit their manuscripts to other journals if they feel that the requested revisions are too extensive. Informants explained:

I don't think [reviewing registered reports is] more work and it's also more satisfying because whatever you give is [in terms of] comments are actually integrated and makes the research better. So in that sense, it's nice. And, you know, the second time around when the full manuscript is there, it's basically only the results and a discussion. (APf_PCI_8)

I mean, it's a bigger ask than reviewing after the study has been conducted in some ways. I mean, you know, I mean, [in case of a traditional manuscript submission], all you have to do is be critical. (…) You don't have to try to fix it or think through what they might have done differently because it's too late. And so this sort of review is, I think, a little more, it takes a little more time. But I do think it makes for better, I think it makes for better science. I mean, we've published several of them, and I've really liked, I've really liked being on the other side of that review process. (Pm_PCI_7)

One could say that the format of registered reports eases tensions between gift-giving in peer review and the competition for reputation by making peer review more akin to co-authorship. This does, of course, raise new questions as to how to distinguish the contribution of a reviewer from that of a formal co-author. It appears that reviewers manage this tension by weighing on a case-by-case basis how far-reaching their input should be. One reviewer explained that they sometimes intentionally avoid suggesting too many improvements in an effort to limit their conceptual involvement in a submission.

[A]nother element that I find, let's say, critical or essential, difficult is that to what extent I should, let's say, help the authors and suggest things and become the major one of the important contributors to the paper. (...) So I try not to go too far with… with suggestions because, yeah, I'm not the author of the research. (AssPm_PCI_11)

While replacing mutual suspicion with a more collaborative relationship between authors and reviewers, the format of registered reports also creates certain new problems. Registered reports can, on the one hand, be seen to promote risky research, insofar as in-principle-acceptance of research designs allows authors to pursue risky questions regardless of the outcomes. One reviewer explained that:

[Registered reports make particular sense] If perhaps [the authors] are asking a risky question or they're doing… they're working in an area of research where there's a lot of publication bias and maybe a lot of suppression of inconvenient results as well.

Yet in-principle-acceptance can also be exploited. One recommender explained their perception that the research designs submitted to PCI:RR are often somewhat unambitious and incremental (PDm_PCIr_16). A reviewer also described an experience where authors proposed a partial replication of a previous study (Pf_PCI_14). While this intended replication appeared methodologically robust, its limited scope robbed it of any intellectual merit. The reviewer recommended not accepting the submission in this form and was frustrated when the recommender decided to accept it regardless. Needless to say that situations in which editors override reviewer recommendations can threaten the commitment of reviewers to the platform if they are encountered too frequently.

To sum up, the publishing model of PCI:RR is comparable to that of Collabra insofar as it effectively eases the tension between gift-giving in peer review and accumulation dynamics in publishing. Its non-commercial business model based on preprints prevents reviewers from having to question their role in the political economy of scholarly publishing, while the relatively lower visibility and prestige of the outlet mitigate the problem of unsuitable and intentionally misleading submissions. The format of registered reports starkly limits the room for questionable author practices and configures peer review more as a form of co-authorship.

## Discussion

The current strain on peer review systems is often framed as a problem of incentives (Zaharie & Seeber 2018; Zollman et al. 2024; see also Waltman et al. 2023). Parts of the literature on scholarly communication and research evaluation, for example, stress primarily how researchers are driven by evaluation requirements, which incentivize them to focus on formally rewarded activities such as publishing and grant-writing (Burrows 2012; Craig et al. 2014). Conversely, critical academic practitioners and activists often call for creating more incentives to take on peer review work, for example, by means of credit systems (Haak & Lawrence 2015; Research Information 2014). Yet while these discourses capture relevant aspects of the problem, they typically operate with somewhat reductive understandings of incentives, either by equating them with formal recognition systems or by focusing only on a single framework of incentives.

In this paper, I have attempted to offer a more complex picture. I have conceptualized engagement in peer review as a gift economy. By this I mean an economic model where resources are allocated on the basis of a logic of mutual obligation, which must be constantly renewed for the continued functioning of a peer review system. The sense of mutual obligation is always threatened by the excesses of accumulation logics, which produce inequities and encourage individualistic behavior that rather undermines the sense of reciprocity. I have proposed that to understand the challenges currently faced by peer review systems, it is useful to compare how publishing outlets handle the inherent conflict between communal labor and the accumulation-based economic systems in which peer review is embedded.

PS presents an example of a journal that has let the resulting tensions go unchecked (at until the recent introduction of various innovations to its publishing workflow). The tensions manifest in two particularly striking ways. First, the journal’s status and traditional publishing model appear to produce tremendous pressure on established gift relations, both in the sense of needing a lot of reviewers and exposing them to often questionable author practices that in turn make them engage in a style of reviewing best described as gatekeeping. Second, the journal relies on a commercial business model that tends to discourage at least some reviewers from donating their time for peer review. This has become empirically visible only in interviews with informants for the other case studies. Some of these explained that they had stopped reviewing for journals with traditional publishing formats, in part because high APCs and subscriptions prices make them question their role in sustaining problematic publisher business models. PS continues to have a functional peer review system in large part because of its still considerable status in the field, which offers reviewers a share of journal prestige that they can in turn use to advance their own competitive position. Naturally, such a system only works for already reputed journals.

Yet review systems are not bounded entities but rather can be seen as corresponding vessels. Negative experiences during peer review for more traditional journals help fuel interest in alternative publishing formats, such as those of Collabra and PCI:RR. Both offer less transferable reputation to begin with, which preemptively avoids issues such as large volumes of misplaced submissions. Yet they also have adopted formal policies that mitigate the problem of questionable submission practices, for example, through mandatory inclusion of research data and the format of registered reports. Both publication venues moreover operate with distinct not-for-profit business models that prevent reviewers from feeling compelled to question the function of their work. Aside from preventive measures to avoid strain on gift relationships, Collabra and PCI:RR also pursue strategies to actively create a sense of mutual obligation among authors and reviewers. I have shown how open peer review and the format of registered reports each encourage constructive interactions, through increased public scrutiny as well as through fostering shared commitment to developing scientific arguments. While this creates some new challenges, it turns reviewing into a somewhat more collaborative process and likely leads to a more sustainable peer review system in the long run. These examples also make clear that engagement around peer review is a distinct factor in the changing social structure of scientific fields. Positive and negative experiences in peer review can lead researchers to abandon particular journals and publishing models in favor of competing options.

The analysis thus complicates the commonsensical notion that formal incentives for individual career development override communal ones per default. Such a view would reduce researchers’ reflexivity about their publishing practices to a mere adaptation mechanism and downplays their agency. Simultaneously, it problematizes the argument that there are no or insufficient incentives for peer review. The work presented here instead suggests that peer review activities are embedded in a surplus of already existing but usually undertheorized incentive systems (see also Zaharie & Osoian 2016). Rather than creating new incentives, we must better balance the dynamics and ripple effects arising from their interactions.

The empirical work presented above will ideally provide inspiration for how to go about this even beyond the case of psychology. Yet it is also clear that many of the strategies I have highlighted are built around a particular epistemological tradition that limits the possibility to directly transpose them to other fields. For example, registered reports and mandatory inclusion of raw data rely on increasing mutual social control among researchers around a shared concern with hypothesis testing through inferential statistics. Proposing them as a model for, say, interpretive scholarship will likely be perceived as unhelpful and even disruptive (Penders et al. 2019). Researchers hoping to tackle structural conflicts between gift and accumulation logics in publishing in other fields must therefore start from the epistemic, social and infrastructural conditions of their respective epistemic culture.

## Footnotes

1 Quotes are referenced via abbreviations that indicate the informant’s career position (PD for postdoc, L for lecturer, AP for associate professor, E for extra-academic etc.), their gender (m/f/x), and the publication outlet on which the interview focused (PS, COL, PCI:RR; followed by a lower case -e for interviews with editors).

2 I am grateful to one of the reviewers of this paper for suggesting the term.
